# The role of Pleistocene climate change in the genetic variability, distribution and demography of *Proechimys cuvieri* and *P*. *guyannensis* (Rodentia: Echimyidae) in northeastern Amazonia

**DOI:** 10.1371/journal.pone.0206660

**Published:** 2018-12-17

**Authors:** Claudia Regina Silva, Camila Cherem Ribas, Maria Nazareth F. Da Silva, Rafael Nascimento Leite, François Catzeflis, Duke S. Rogers, Benoit De Thoisy

**Affiliations:** 1 Programa de Pós-Graduação em Genética, Conservação e Biologia Evolutiva, Instituto Nacional de Pesquisas da Amazônia, Petrópolis, Manaus, AM, Brazil; 2 Laboratório de Mamíferos, Instituto de Pesquisas Científicas e Tecnológicas do Estado do Amapá (IEPA), Macapá, AP, Brazil; 3 Programa de Coleções Científicas Biológicas, Instituto Nacional de Pesquisas da Amazônia, Petrópolis, Manaus, AM, Brazil; 4 Institut des Sciences de l’Evolution, CNRS UMR-5554, Université Montpellier-2, Montpellier, France; 5 Monte L. Bean Life Science Museum, Brigham Young University, Provo, UT, United States of America; 6 Institut Pasteur de la Guyane, Cayenne cedex, French Guiana; 7 Association Kwata, Cayenne, French Guiana; National Cheng Kung University, TAIWAN

## Abstract

The spiny rats, genus *Proechimys*, have the highest species richness within the Echimyidae family, as well as species with high genetic variability. The genus distribution includes tropical South America and Central America south to Honduras. In this study, we evaluate the phylogeographic histories of *Proechimys guyannensis* and *P*. *cuvieri* using cytochrome b, in a densely sampled area in northeastern Amazon where both species are found in sympatry in different environments. For each species, Bayesian and Maximum Likelihood phylogenetic analysis were congruent and recovered similar clades in the studied area. Bayesian phylogenetic analysis using a relaxed molecular clock showed that these clusters of haplotypes diversified during Pleistocene for both species. Apparently, the large rivers of the region did not act as barriers, as some clades include specimens collected from opposite banks of Oiapoque, Araguari and Jari rivers. Bayesian skyline plot analysis showed recent demographic expansion in both species. The Pleistocene climatic changes in concert with the geologic changes in the Amazon fan probably acted as drivers in the diversification that we detected in these two spiny rats. *Proechimys cuvieri* and *P*. *guyannensis* show genetic structure in the eastern part of the Guiana region. Greater genetic distances observed in *P*. *guyannensis*, associated with highly structured groups, suggest that more detailed studies of systematics and ecology should be directed to this species.

## Introduction

The considerable biological diversity of the Amazon region has long stimulated the interest of researchers in relation to species richness [1, 2, 3], diversification process [4, 5, 6] and endemism [7, 8, 9]. The patterns of species distribution and genetic variability are complex in the Amazon, varying between taxa with distinct responses to historical events [10, 11, 12]. Wallace [7] was the first naturalist to observe that major rivers could represent barriers between populations separated in different margins, promoting allopatric speciation. The so-called riverine barrier hypothesis has been corroborated by a number of studies, in particular with birds and primates [5, 13, 14, 15], but this model was not upheld for other mammalian taxa [4, 10, 16, 17] or frogs [12, 18]. Leite & Rogers [19] postulated that the effect of a river as a barrier depends largely on the vagility of the focal organism and that is conditioned to the geography and historical formation of the drainage system. An alternative hypothesis invoking Pleistocene refugia postulates that climatic oscillations during this period modified patterns of vegetation cover and the degree of landscape connectivity, promoting isolation among populations [8, 20]. However, palynological and geomorphological studies [21] and climate modeling [22] suggested that vegetational changes in the Quaternary were not enough to fragment the Amazon forest into a mosaic of forest fragments isolated by open vegetation.

In small non-volant mammals, phylogeographic structure and genetic diversification may have been influenced by historical events such as the establishment of the rivers [19, 23], geological formations like the Iquitos arch [10] and changes in vegetation [11]. For the full understanding must also require considering the ecology and life history strategies of the species [16, 17]. Studies evaluating phylogeographic patterns in small, non-flying mammals suggested that there is no consensus and that the interaction between different events is to be expected [4, 11, 24, 25]. Bonvicino & Weksler [25] postulated that the mammalian fauna of the Amazon is still sub-sampled and the spatial and temporal genetic patterns of most of its organisms are little known.

**Fig 1:**
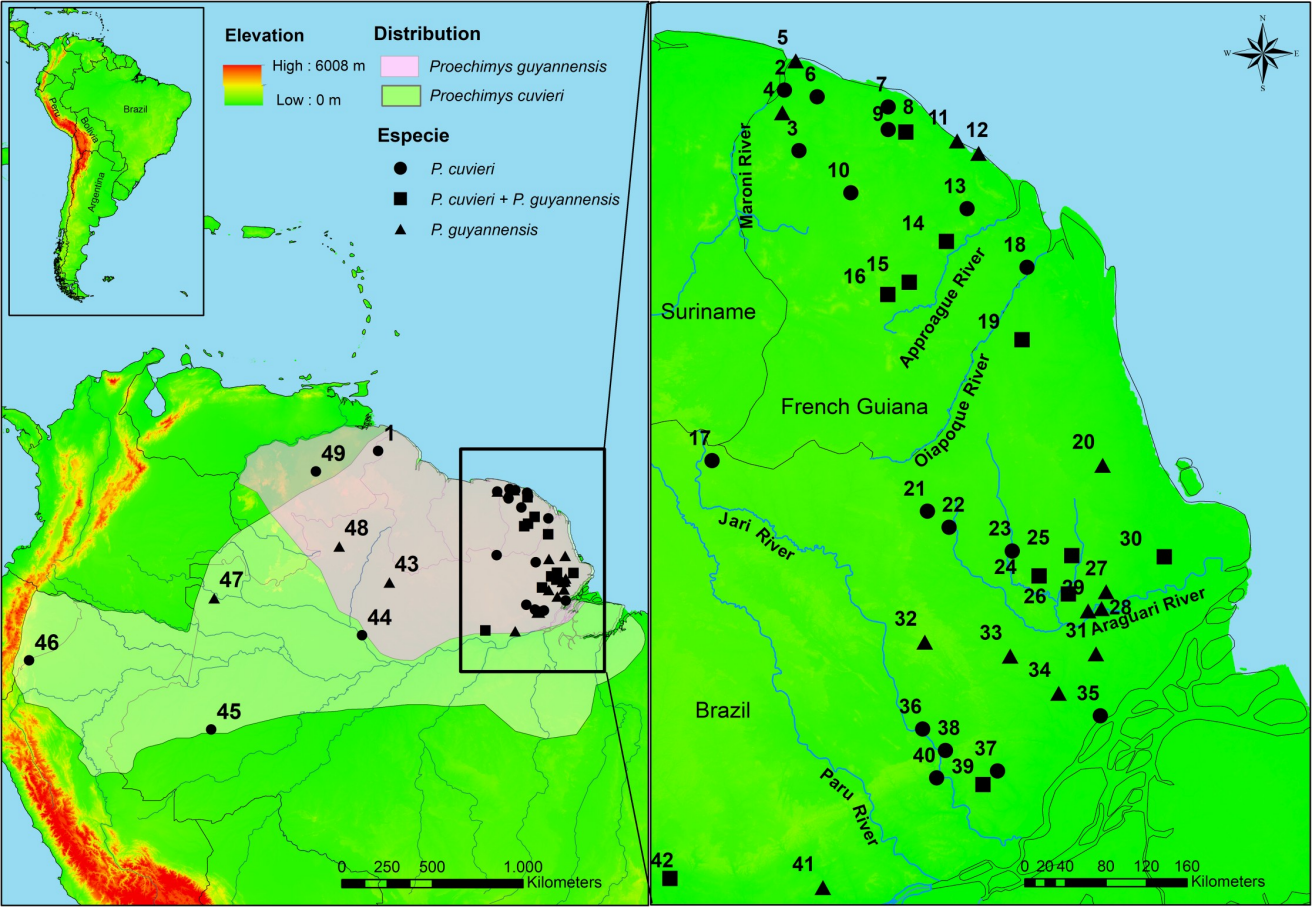
Locations of *Proechimys cuvieri* and *Proechimys guyannensis*. **Distribution of the species are identified in light green for *P*. *cuvieri* and in salmon for *P*. *guyannensis*.** The numbers identify the locality of the samples: (1) Baramita, (2) Ste Anne, (3) St Jean, (4) Sparouine, (5) Awala, (6) Angoulème, (7) Piste St Elie, (8) Sinnamary, (9) Petit Saut, (10) Trinité, (11) Macouria, (12) Cayenne, (13) Piste Belizon, (14) Nouragues, (15) Pic Matecho, (16) Saül, (17) Mapaone, (18) Saint Georges, (19) Anotaie river, (20) Amapá Grande river, (21) Anacuí river, (22) Amapari river, (23) Mutum river, (24) Santo Antonio stream, (25) Braço stream, (26) Falsino river, (27) Tracajatuba river, (28) Ferreira Gomes, (29) Caldeirão Falls, (30) São Bento Farm, (31) Horto Matapi, (32) Cupixi river, (33) Vila Nova river-forest, (34) Vila Nova river-savana, (35) Santana Island, (36) Jari river, (37) Marinho Village, (38) Itacará, (39) Santo Antônio Falls, (40) Porto do Sabão, (41) Jatuarana Village, (42) Mamiá Village, (43) Alto Jatapu, (44) Meduinim Lake, (45) Barro Vermelho, (46) Pico da Neblina, (47) Amazonas, (48) Serra do Apiaú, (49) Bolívar.

*Proechimys* spiny rats comprise the most species-rich genus within the family Echimyidae. The genus distribution includes tropical South America and Central America south from Honduras [26]. These terrestrial rodents are also characterized by considerable genetic and morphological variation found both within and between populations, which has hindered taxonomic studies and the reliable delimitation of geographic ranges [26, 27, 28]. Twenty-two species arranged in 10 species groups are currently recognized for the genus *Proechimys*. Two species occur in sympatry in the northeastern Amazon basin: *Proechimys cuvieri* Petter, 1978 is monotypic and part of the *longicaudatus* species group [26]; and *Proechimys guyannensis* (E. Geoffroy, 1803) is also considered monotypic (despite high intraspecific variation; see comments in Patton & Leite, [26]) and belongs to the *guyannensis* species group. Both species are characterized by considerable karyotypic and molecular variability [28, 29, 30]. *Proechimys cuvieri* has a wide distribution (Fig 1), occurring along both margins of the Amazon (Solimões) river from Peru to its mouth, as well as throughout the entire region of the Guianas [26]. The distribution of *P*. *guyannensis* is restricted to the Guianas (Fig 1) [26, 27].

Patton et al. [10] identified four distinct clades in *P*. *cuvieri* based on the mitochondrial cytochrome b (cytb) gene. These clades share basic morphological traits, like skull and extern morphology, and have karyotypes with the same diploid number but different fundamental numbers [30, 31, 32]. One of the clades is distributed throughout the Guianas and east of the lower Negro River and both banks of the lower Amazon River. *Proechimys guyannensis* has high levels of karyotypic and molecular variability [27, 28, 32, 33]. Bonvicino et al. [28] identified distinct karyotypes in *P*. *guyannensis* from the Brazilian state of Amazonas and suggested that these represented distinct evolutionary lineages. Based on cytb sequences from a number of different localities in French Guiana, Steiner et al. [29] and Van Vuuren et al. [34] showed that *P*. *cuvieri* is characterized by higher diversity indices than *P*. *guyannensis*, verified by the number of informative sites and nucleotide diversity. Van Vuuren et al. [34] suggested too that the populations of the *P*. *guyannensis* that have recently colonized new regions and/or are expanding. *Proechimys guyannensis* and *P*. *cuvieri* are recorded frequently in inventories of small non-volant mammals in the eastern Guianas, in particular within the area between the left margin of the Amazon River in the Brazilian state of Amapá and the Maroni River, which forms the border between French Guiana and Suriname [29, 35]. These terrestrial spiny rats are found in a range of habitats, including *terra firme* forests, flooded forests, forest-fragments in savannahs [36, 35], in gallery forests and in isolated tracts of forest within marshland. *Proechimys guyannensis* and *P*. *cuvieri* are found in sympatry and syntopy in French Guiana [29] and other locations in the Amazon [36, 37].

In the present study, we used the mitochondrial cytb gene to characterize the intraspecific genetic diversity of *P*. *guyannensis* and *P*. *cuvieri* in the eastern portion of the Guiana region. Cytb has been widely used in studies of genetic variability and phylogenetics of small mammals [38, 39]. Although mutation rate is high in mitochondrial DNA, it is a largely useful molecular tool for the reconstruction of the history of populations and species due to its relatively simple amplification, typically non-recombinant haploids, and its high degree of intra and interspecific variability [40, 41].

Here, relying on extensive surveys and sampling in the northeastern Amazon basin, we evaluate the demographic history and phylogeography of the two species *Proechimys guyannensis* and *P*. *cuvieri* and aim to describe the factors that have determined the patterns of genetic variability in the context of the geological and climatic history of the region.

## Materials and methods

### Study area

Tissue samples used in this study were distributed from French Guiana and the Brazilian states of Amapá and Pará, situated in the eastern portion of the Guiana region. This region is well-preserved, with large areas of forest protected by a network of conservation units on both sides of Brazil and French Guiana [42, 43, 44]. The main forest areas are found in the western portion of the study area, located on substrates of Precambrian origin [45]. Isolated savannahs are found throughout the study area [46] located between the forests of the interior portion and the coastal region. The coastal zone of southern Amapá is influenced primarily by the adjacent Amazon River, which forms extensive areas of seasonally flooded habitats, with many lakes and islands [47]. The northern coast of the state, north of the Araguari River to French Guiana, encompasses tracts of mangroves interspersed with flooded fields influenced by the Atlantic Ocean (Fig 1). The Brazilian portion of the study area is the most impacted, with extensive deforestation and human colonization along the Atlantic coast and the northern margin of the Amazon River in Pará State [48]. A more recent impact is the ongoing expansion of soybean plantations into the savannah habitats of Amapá State [46, 49]. In French Guiana, the principal threats are mining [50, 51], and deforestation, principally on the coast [43], although forest loss remains below the rates observed in other Amazonian countries [52].

### Samples

All tissues samples used in this study were obtained from voucher specimens of the Collection of Amapá Fauna of the Institute for Scientific and Technological Research of the state of Amapá (IEPA) in Macapá; National Institute of Amazon Research (INPA) in Manaus (both in Brazil), and Collection JAGUARS, Association Kwata and Institut Pasteur de la Guyane, Cayenne. All analysed samples were obtained specimens deposited in the aforementioned scientific collections, thus no collecting permits were required. Catalog numbers are given in S1 Appendix. INPA´s Ethics Committee on Animal Use (CEUA) of INPA granted formal waiver of ethics approval due to that tissue of the sequences we generated were obtained from scientific collections.

We sequenced 1100 base pairs (bp) of the CYTB gene in 37 samples of *Proechimys cuvieri* from 22 localities and 54 samples of *P*. *guyannensis* from 20 localities within the study area, between northern French Guiana and Brazil, north of the Amazon River (Amapá and Pará). The two species were sympatric at 11 sites (Fig 1). We also sequenced 800 bp of CYTB in 8 samples collected in Brazil (Amazonas and Roraima states), Venezuela, and Peru (Fig 1) for phylogenetic analyses. Only 25 additional sequences were obtained from GenBank. We analyzed a total of 124 sequences of *P*. *cuvieri* and *P*. *guyannensis* in 50 localities; details on specimens, localities and GenBank accession numbers, are given in S1 Appendix.

### Sequencing

The DNA was extracted from tissue samples, obtain of the muscle and skin byopsy, preserved in 95% ethanol. The DNA was extracted using the NucliSENS EasyMag robot (Biomérieux, Craponne, France) and Wizard Genomic DNA Purification (Promega) kits, according to the manufacturers’ protocols. Three overlapping fragments of the cytb were amplified using internal and external primers. We amplified the first fragment, of 400–500 bps, using the primers H6 (5'TCTTCCATGAGGACAAATATC3') and L15 (5’TCTCCATTTCTGGTTTACAAGAC3'), and the final fragment (also 400–500 bps) using the primers H8 [28] and L2 (5' TACCATGAGGACAAATATC’). The intermediate fragment, with approximately 400 bps, was amplified using the primers PRO 197F (5´TTACACAYATTTGYCGAGAYG3´) and PRO 665R (5´GGGTGRAATGGRATTTTGTCTGA3´), which were designed specifically for this study. The samples were amplified in 40 μL reaction volumes using Platinum Taq (Life Technologies Corp.) containing 4.0 μL of each primer, 5.0 μL of reaction buffer, 3.0 μL of MgCl^2^ 50 mM, 5.0 μL of dNTP mix, 0.5 μL of polymerase, 2.0 μL of the DNA template, and 16,5 μL of H_2_O. The polymerase chain reaction (PCR) cycle consisted of initial denaturation at 94°C for 3 min, followed by 30 amplification cycles of denaturation at 94°C for 30 sec, annealing at 45°C for 1 min and extension at 72°C for 2 min, with a final extension at 72°C for 10 min.

The PCR products were sent to Beckman Coulter Genomics (Takeley, UK) for purification and sequencing. The sequences were aligned and checked manually using MEGA 6.0 [53]. We did not observe stop codons, and transition and transversion rates were normal.

### Analyses

We implemented Bayesian Inference (BI) and Maximum Likelihood (ML) phylogenetic analysis to reconstruct the relationships within the studied populations of *P*. *guyannensis* and *P*. *cuvieri* from northeastern Amazonia using the complete dataset. Sequences of the rodent species *Trichomys apereoides* (AY083332, AY083341) and *Hoplomys* (HM544128, NC020657) were obtained from GenBank and included in the analyses as outgroups.

The program jModelTest version 3.7 [54] was used to select the HKY+G like the best evolutionary model for the datasets based on Bayesian Information Criterion (BIC). The BI phylogenetic tree was reconstructed in MrBayes 3.2.1 [55], using 10^7^ Markov Chain Monte Carlo (MCMC) generations, with the first 25% being discarded as burn-in. The ML analysis was run in RAxML in the CIPRES Science Gateway [56]. Nodal support was estimated with 100 bootstrap replicates. We estimated the divergence time between the lineages using the relaxed lognormal uncorrelated molecular clock and the Yule Process pure-birth speciation model, run in BEAST 1.6.2 [57]. The substitution rate used in the analysis was the 3.6% divergence per million year, derived from Fabre et al. [58]. We used the following times inferred from Fabre et al. [59]: divergence between [*Trichomys*] and [*Hoplomys* + *Proechimys* spp.] set at 9.4 Ma (95% CI: 8.3–10.9) and divergence between *Hoplomys* and *Proechimys* set at 5.5 Ma (95% CI: 4.6–6.7), with these dates being used as calibration for the most recent common ancestor (TMRCA), considering a normal distribution. Since no more recent calibration point was available, we confirmed the absence of substitution saturation on the [*Trichomys* + *Hoplomys* + *Proechimys*] dataset with the Xia test [60] implemented with DAMBE [61]. We generate 10^7^ Markov Chain (MCMC) generations, with the first 25% being discarded as burn-in. We used TRACER 1.6 to evaluate the convergence of the analysis and the parameters estimated, considering an Effective Sample Size (ESS) equal to or greater than 200 [62].

We generated haplotype networks using Network 4.6 and the median-joining (MJ) method [63]. BAPS5 (Bayesian Analysis of Population Structure, [64]) was employed to verify population structure. We ran the mixture model setting the maximum possibility of clusters [64]. The genetic distance between clusters identified in BAPS5 and between clusters composed for sequences with distribution between the rivers (Oiapoque–Maroni; Araguari–Oiapoque, Jari–Araguari, and Jari–Trombetas) (Fig 1). Genetic distance was calculated using MEGA 6.0 [53].

We considered the isolation effect of different river drainages to evaluate gene flow patterns between geographic areas. We used the program Migrate [65] under a Bayesian framework to estimate effective population sizes (Θ) and migration rates M between different populations from north to south in studied area. We ran four chains with thermodynamic integration and a static heating swap scheme (temperatures: 1.0, 1.5, 3.0, 10^6^), sampling at every 100^th^ step for a total of 20^6^ steps and a burn-in of 50^3^ steps.

We calculated Fu’s Fs [66], Tajima’s D [67] and R2 [68] to assess potential deviations from neutrality. We used the program DNASP version 5.0 [69] and significance was determined from 10,000 coalescent simulations. For each *Proechimys* species, we analyzed the complete dataset, the clusters from BAPS analyses, and samples separated by main interfluves (Maroni–Oiapoque; Oiapoque–Araguari; Araguari–Jari; Jari–Trombetas). We used the Mismatch Distribution Analysis [70] with the sum of square deviations (SSD) between the observed and the expected mismatch and the ragggedness index as statistic tests and generated a Bayesian Skyline Plot (BSP) in BEAST 1.6.2 [71] to assess the demographic history of the clusters for both species over time. We only inferred BSP for clusters with more than 10 sequences. We used HKY+G evolutionary model and Yule prior. We ran 10^7^ MCMC generations, excluding the first 25% samples as burn-in. Effective sample sizes, parameter estimates, and convergence of the Markov chains were determined using TRACER 1.6 [62].

## Results

### Phylogenetic relationships in *P*. *guyannensis* and *P*. *cuvieri*

**Fig 2:**
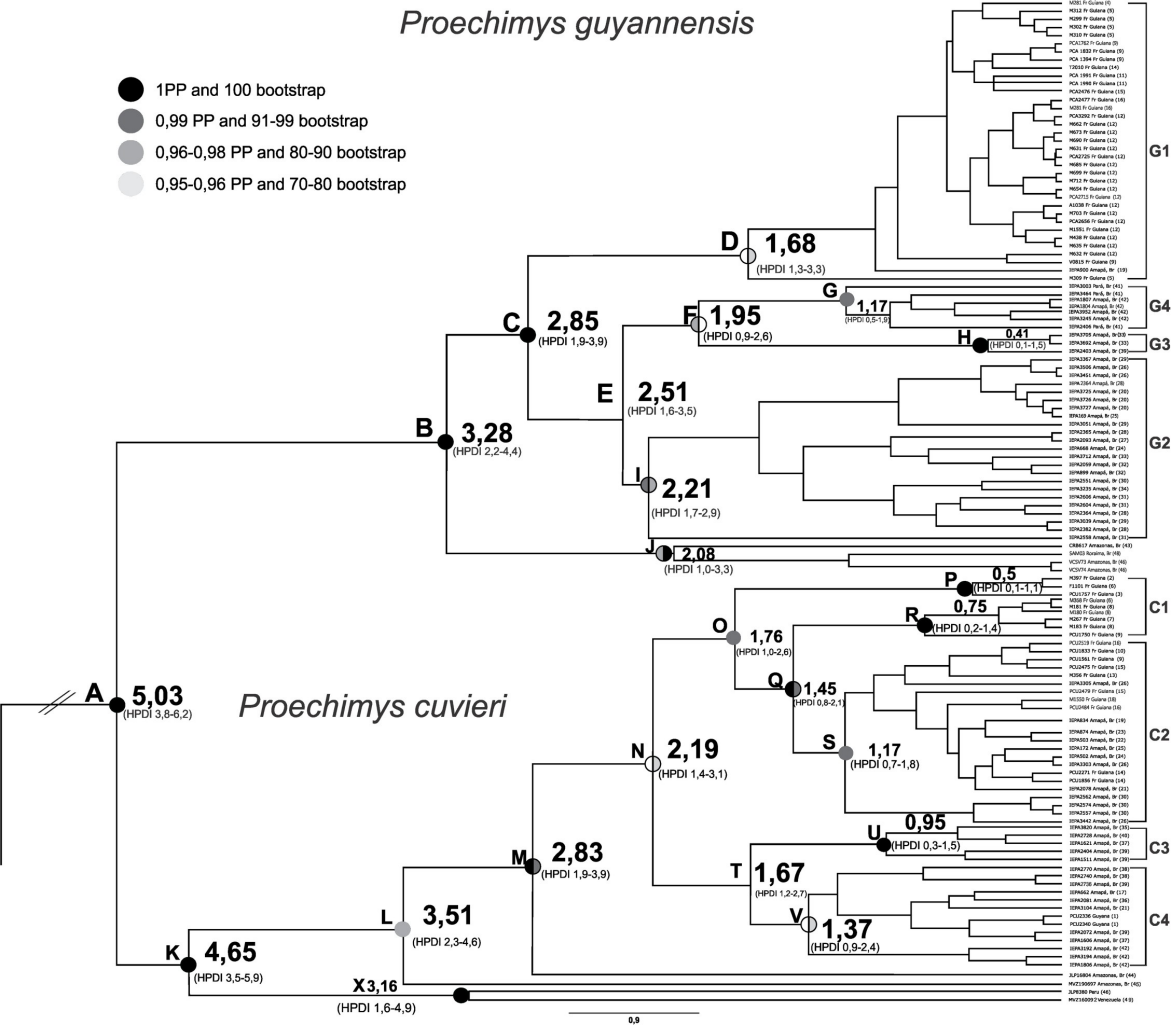
Molecular dating chronogram using cytochrome b with a HKY + I + G model (ML and BI topology presents similar topology). **Statistical support higher than 0.90 posterior probability (PP) and 70 bootstraps is identified next to the nodes divided in two sides, the left side represents PP values and the right side the bootstrap values; black represent 1 PP and 100 bootstrap, very dark gray represent 0,99 PP and 91–99 bootstrap, dark gray represent values between 0.96–0.98 PP and 80–90 bootstrap, and light gray represents values between 0.95–0.96 PP and 70–80 bootstrap.** The letters B, C, D, E, F, G, H, J and I identify clades formed in *P*. *guyannensis*. The letters K, L, M, N, O, P, Q, R, S, T, U, V and X identify clades formed in *P*. *cuvieri*. Each terminal is identified for sample followed by country of origin. The numbers in the parentheses identified the locality in the Fig 1. We also identified the state of origin in the case of Brazil. Clusters recovered by BAPS are identified after the terminals for *P*. *guyannensis* (G1, G2, G3, G4) and for *P*. *cuvieri* (C1, C2, C3, C4). Inferred spliting dates are shown on the nodes.

The phylogenetic reconstruction presented similar results in BI and ML analyses (Fig 2, see too S2 Appendix and S3 Appendix). Our data for the eastern portion of the Guianas region also formed monophyletic clades for both *P*. *guyannensis* (clade B) and *P*. *cuvieri* (clade K). The phylogenetic reconstruction identified *P*. *guyannensis* clades distributed between the north and southeast of the study area (clade C, Fig 2). The samples from Amazonas, Roraima, and Venezuela formed clade J. In clade C, the samples were organized in four subclades: the haplotypes from French Guiana and northern Amapá (clade D), central Amapá (clade I), south-east Amapá (clade H) and two localities in Pará (clade G). These clades were well-supported statistically either with bootstraps (ML) or posterior probabilities (Bayesian analysis), with the exception of clade D, which was supported only weakly by the ML analysis (Fig 2). Five clades were evidenced in *P*. *cuvieri* (clade N, Fig 2), two in northern French Guiana (clades P and R), one that groups haplotypes between central northern French Guiana to the Araguari River in Amapá (clade S), south-east Amapá and Pará, including the sample from Baramita, Guyana (clade V) and haplotypes from the Jari River, associated with those from Santana Island, in the Amazon Estuary (clade U). These clades were supported statistically by the PP or bootstrap analysis (Fig 2).

We infer that *P*. *guyannensis* and *P*. *cuvieri* diverged in the Pliocene, around 5.03 million years ago (Mya), with 95% confidence of Highest Posterior Density Interval (HPDI) ranging between 3.8–6.2 Mya. The diversification of the clades found in the eastern portion of the Guiana region occurred in late Pliocene, at around 2.85 Mya (95% HPDI: 1.9–3.8 Mya) in *P*. *guyannensis* and more recently in early Pleistocene in *P*. *cuvieri*, at 2.19 Mya (95% HPDI: 1.4–3.1 Mya).

### Genetic structure of the populations

**Fig 3:**
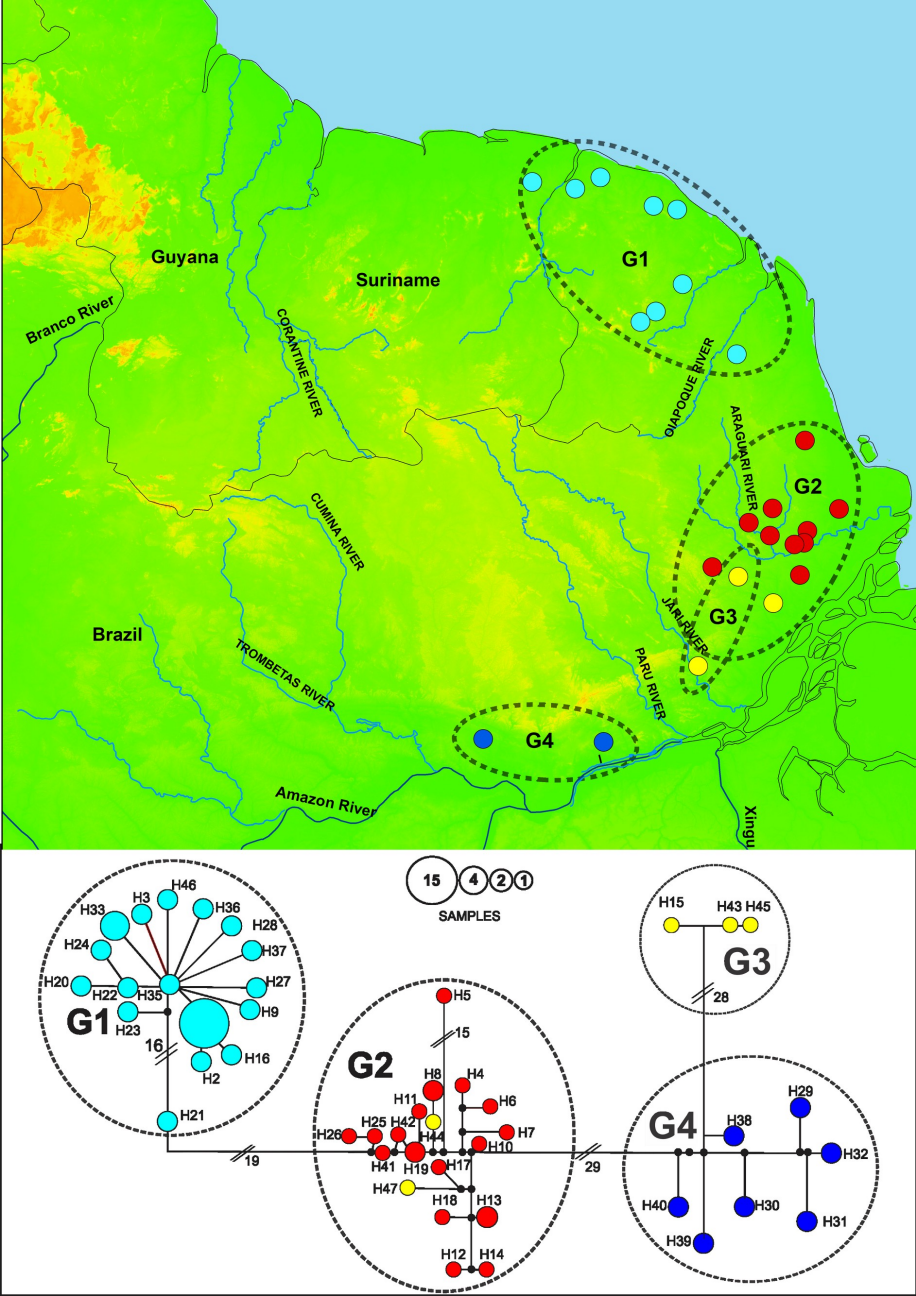
**Map (top) showing the locations of the *P*. *guyannensis*, with circle colour show the regions within the study area: North, from the Oiapoque river basin to the Maroni River (light blue); Centre, Araguari River basin (red); Southeast, included Jari and Vila Nova river basins (yellow); and Mamiá and Jatuarana villages, state of Pará (blue)**. Based on cyt-b the haplotype network (bottom) has branch lengths corresponding to the nucleotide substitutions. The number of mutational steps of the largest branches is shown under the branches. Circle sizes correspond to the number of individuals sharing the same haplotype. Circle colors correspond to the colors of the regions identified on the map. Circles in black correspond to the mean vectors. The clusters recovered by BAPS are outlined and identified as G1, G2, G3 and G4 on the map and haplotype network.

Both *Proechimys* species had high haplotype diversity and low nucleotide diversity (Table 1). In *P*. *guyannensis*, the 67 sequences analyzed provided 43 haplotypes, 155 polymorphic sites, and 101 informative sites for parsimony analysis. In *P*. *cuvieri*, the 49 sequences analyzed returned 45 haplotypes, 140 polymorphic sites, and 94 informative sites. Once analyzed with BAPS, the haplotypes of *P*. *guyannensis* were recovered in four clusters (G1, G2, G3 and G4). These clusters correspond to the clades recovered in the phylogenetic tree (Fig 2). The haplotypes in the northern part of the study area, including samples from French Guiana and one location in the state of Amapá on the Anotaie River, a tributary of the Oiapoque, formed cluster G1. The haplotypes from the south-east of the study area, from the localities on the Jari and upper Vila Nova rivers formed group G3. Group G4 brings together the samples from two localities between the Jari and Trombetas rivers (Fig 2). The haplotype network recovered the clusters identified by the BAPS, resulting in four clusters separated from each other by more than 20 mutational steps. The geographical layout encompasses clusters distributed from north to south. Significant haplotype sharing was observed in the Cayenne locality (point 12 in Fig 1) in the cluster G1, fifteen samples shared haplotype 1 (H1, Fig 3). Although this pattern was not upheld in other clusters which were characterized by limited haplotype sharing.

**Table 1:** Haplotype diversity (HD), nucleotide diversity (π) and the number of individuals (N) in the clusters identified by the BAPS for *P*. *guyannensis* (G1, G2, G3, G4) and *P*. *cuvieri* (C1, C2, C3, C4), the interfluves of the study region: Maroni-Oiapoque; Oiapoque-Araguari; Araguari-Jari e Jari-Trombetas and the complete dataset for each species.

*P*. *guyannensis* Cluster	Hd	π	N	*P*. *cuvieri* Cluster	Hd	Π	N
**G1**	0.7660	0.00416	35	**C1**	0.9333	0.01523	9
**G2**	0.9860	0.01358	21	**C2**	0.9800	0.01089	22
**G3**	0.6667	0.00239	3	**C3**	1.0000	0.01023	5
**G4**	1.0000	0.01225	7	**C4**	1.0000	0.02015	10
**Oiapoque-Maroni**	0.7524	0.00391	36	**Oiapoque-Maroni**	0.9819	0.01697	22
**Oiapoque-Araguari**	0.9714	0.01453	15	**Oiapoque-Araguari**	0.9487	0.01122	11
**Araguari-Jari**	0.9818	0.02175	11	**Araguari-Jari**	1.0000	0.01868	12
**Jari-Trombetas**	1.0000	0.01225	7	**Jari-Trombetas**	1.0000	0.01399	4
**All samples**	0.921	0.02378	67	**All samples**	0.9935	0.02015	49

**Fig 4:**
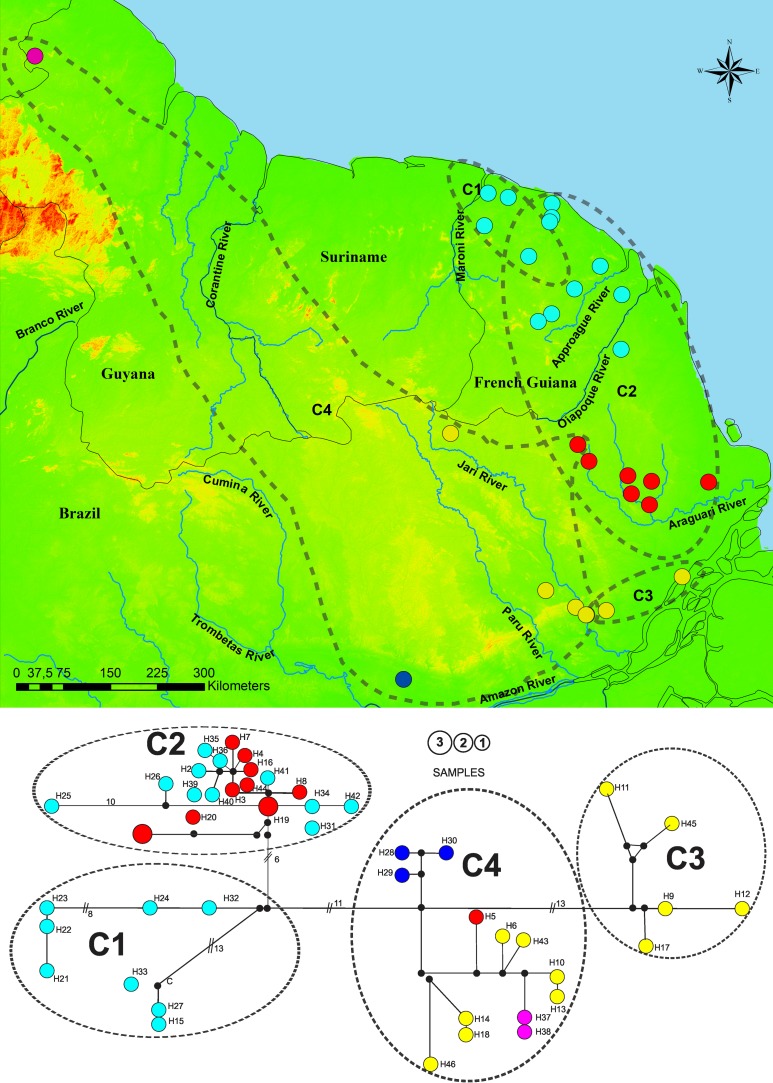
**Map (top) showing the locations of *P*. *cuvieri*, with circle colour showing the regions within the study area: North, from the Oiapoque River basin to the Maroni River (light blue); Centre, Araguari River basin (red); Southeast, included Jari and Vila Nova River basins (yellow); and Mamiá and Jatuarana villages, state of Pará (blue).** Based on cyt-b the haplotype network (bottom) has branch lengths corresponding to the nucleotide substitutions. The number of mutational steps of the largest branches is shown in the figure captions. Circle sizes correspond to the number of individuals sharing the same haplotype. Circle colors correspond to the colors of the regions identified on the map. Circles in black correspond to the mean vectors. The clusters recovered by BAPS are outlined and identified as C1, C2, C3 and C4 on the map and haplotype network.

The BAPS recovered four *P*. *cuvieri* clusters (C1, C2, C3 and C4), matching the clades recovered in the phylogenetic tree (Fig 2). Except the clades P and R (Fig 2) which make up cluster C1 in the BAPS results. The C1 cluster includes the haplotypes from the northern-most area within the study region, between the Maroni and Sinnamary rivers in French Guiana. The haplotypes distributed between central French Guiana and central Amapá, north of the Araguari River, form cluster C2. Haplotypes from the south-east region were recovered in a cluster together with a haplotype from Santana Island, in the Amazon Estuary formed cluster C3. Finally, the C4 cluster linked the samples from the Mamiá, a tributary of the Curuá River, with haplotypes from the Jari River and Baramita, in Guyana (Fig 4). The haplotype network also presented a topology similar to that of the haplotype tree, although the two clades P and R formed a single cluster (C1), in agreement with the BAPS results. The *P*. *cuvieri* clusters were separated from each other by fewer mutational steps, between six and 13. There was relatively little sharing of haplotypes, and a large number of unique haplotypes (Fig 4).

We found the largest intraspecific genetic distances for both species when we compared the clusters identified by the BAPS, with p-distance ranging from 3.35% to 4.23% (Table 2) in *P*. *guyannensis*. Smaller values (0.84–1.48%) were found when comparing the interfluves (Maroni-Oiapoque, Oiapoque-Araguari, Araguari-Jari, Jari-Trombetas). Similarly, while we found high genetic distance values between interfluvial regions in *P*. *cuvieri* (p-distances: 1.2–2.53%), they were still lower than those recorded between clusters (1.84–3.17%; Table 2). Intraspecific genetic distances were smaller in interfluves comparisons than in inter-clusters comparisons. This is well-marked in *P*. *guyannensis* (p-values <0.01 in t-tests and Mann-Whitney tests) but less pronounced in *P*. *cuvieri* (p-values 0.08 in t-tests; not significant in Mann-Whitney test).

**Table 2:** Matrix of the genetic distances (p-distance), based on the sequences of the cytb gene. We compare values from the clusters defined by the BAPS: *P*. *guyannensis* (G1, G2, G3, G4) and *P*. *cuvieri* (C1, C2, C3, C4). We compare between the interfluves of the study region: Maroni-Oiapoque; Oiapoque-Araguari; Araguari-Jari e Jari-Trombetas.

*P*. *guyannensis*				*P*. *cuvieri*			
Cluster	G1	G2	G3	Cluster	C1	C2	C3
**G1**				**C1**			
**G2**	3.35			**C2**	1.84		
**G3**	3.55	3.75		**C3**	3.04	3.17	
**G4**	4.23	3.90	3.54	**C4**	2.52	2.39	2.41
**Interfluves**	**Maroni Oiapoque**	**OiapoqueAraguari**	**Araguari Jari**	**Interfluves**	**Maroni Oiapoque**	**Oiapoque Araguari**	**Araguari Jari**
**Maroni Oiapoque**				**Maroni Oiapoque**			
**Oiapoque Araguari**	1.30			**Oiapoque Araguari**	2.06		
**Araguari Jari**	1.48	0.84		**Araguari Jari**	2.02	2.53	
**Jari-Trombetas**	1.17	1.18	1.37	**Jari-Trombetas**	2.11	2.21	1.20

### Gene flow between populations

We recorded two-way migrations in all regions analyzed. The largest number of migrants (195.40) in *P*. *guyannensis* was recorded from the central region (Araguari basin) to the South region (Jari River, the Amazon and Pará rivers). The second greatest number of migrants (82.60) was recorded between the North region (Oiapoque River and Maroni River), and the South. The lowest values were recorded from the Central region to the North with number of migrants varying between 21.70 and 50.70 (Table 3). In *P*. *cuvieri*, the largest numbers of migrants moved from the North to the Central region, and vice versa. The numbers of migrants recorded between the other regions were much smaller, and well balanced around 60.00 (Table 3).

**Table 3:** Estimated migration among *Proechimys guyannensis* and *P*. *cuvieri* populations, based on analysis using the Migrate software. **The samples were divided into three regions, the North (from the Oiapoque River to Guyana), Central (Araguari basin in Amapá) and South (Amazon Estuary, Jari and Pará rivers).** The plus sign (+) after the name of the region indicates a population receiving immigrants. The highest migration rates are marked in bold type.

Cluster	*P*. *guyannensis*			*P*. *cuvieri*		
	North+	Central+	South+	North+	Central+	South+
**North**		21.7	**82.60**	-	**258.00**	59.90
**Central**	33.70		**195.40**	**620.00**		59.10
**South**	46.50	50.70		58.80	61.90	

### Demographic history

We obtained significant results for the R2 (-2.3781), D (-2.3781), and Fs (-4.572) parameters for the *P*. *guyannensis* cluster G1. When we analyzed the whole dataset, however, no significant values were obtained, although a significant R2 value was obtained for cluster G4 (Table 4). We obtained similar results when we analyzed the data organized by interfluve, with significant values being recorded for all the parameters for the Oiapoque-Maroni interfluve (Fs = -4.168; D = -2.3200; R2 = -0.0617), which has haplotypes similar to the G1 cluster.

**Table 4:** The values of R2 [68], Fs [66], and D [67] for the clusters recuperated by the BAPS (*P*. *guyannensis*: G1, G2, G3, and G4; and *P*. *cuvieri*: C1, C2, C3, and C4), and for the principal interfluves in the study area (samples between Oiapoque and Maroni rivers, between Oiapoque and Araguari rivers; Araguari and Jari rivers and Jari and Trombetas rivers), and the complete dataset.

	*P*. *guyannensis*					*P*. *cuvieri*		
Samples		R2	Fs	D	Samples	R2	Fs	D
**Cluster**	**G1**	**0.05**	**-4.57**	**-2.38**	**C1**	**0.089**	2.12	1.49
	**G 2**	0.092	-3.87	-1.31	**C2**	0.10	**-5.22**	-0.74
	**G 3**	0.47	NA	NA	**C3**	**0.12**	-0.16	-0.84
	**G 4**	**0.11**	-1.0	-0.40	**C4**	0.11	**-4.39**	-0.73
**Between rivers**	**Oiapoque-Maroni**	**0.06**	**-4.17**	**-2.32**	**Oiapoque-Maroni**	0.12	-2.19	-0.11
	**Oiapoque-Araguari**	0.10	-0.58	**-1.50**	**Oiapoque-Araguari**	0.12	-0.33	-0.50
	**Araguari-Jari**	0.17	**-**0.23	0.51	**Araguari-Jari**	**0.10**	**-3.11**	-0.94
	**Jari-Trombetas**	**0.11**	-1.03	-0.40	**Jari-Trombetas**	0.33	0.86	-0.11
	**All samples**	0.08	-3.90	-0.72	**All samples**	0.07	**-16.85**	-1.11

**Fig 5:**
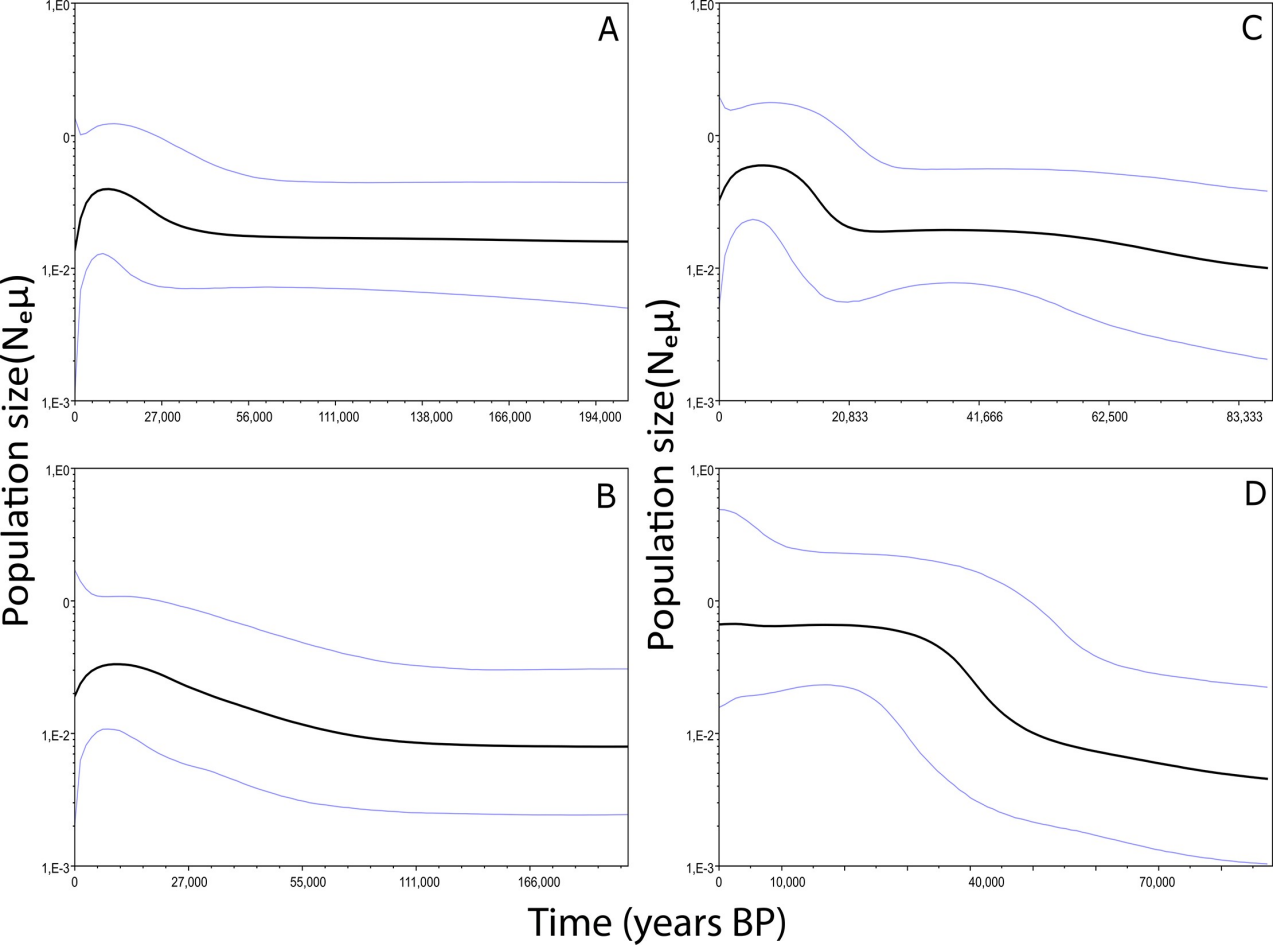
Bayesian Skyline Plots showing the demographic evolution for the *P*. *guyannensis*, G1 (A) and G2 (B) and *P*. *cuvieri* C2 (C) and C3 (D).

In the complete *P*. *cuvieri* dataset, the only deviation from neutrality was found in Fu’s Fs (Table 4). In the analysis of the clusters, we recorded significant Fs values for clusters C2 and C4, and significant R2 values for clusters C1 and C3. In the analysis of the interfluves, we recorded significantly R2 and Fs values for the Araguari-Jari interfluve (Table 4). The Mismatch distribution suggest a significant expansion for G1 only (both SSD and Raggedness tests *p*<0.05), and to a less extent for G2 (Raggedness *p*<0.05, SSD *p* value = 0.08) and G4 (SSD *p*< 0.01). Bayesian reconstruction of the demographic history revealed an expansion event for both species. In *P*. *guyannensis*, the most recent expansion was recorded for cluster G1 and important expansion (revealed by all statistical tests), occurring over the past 30,000 years, whereas in cluster G2, expansion was either quite older and/or of a lower extent (Fig 5). Signals of expansion population were also found in two clusters analyzed of the *P*. *cuvieri*, although they benefited from a lower statistical support (Fig 5).

## Discussion

### Diversification of *P*. *guyannensis* and *P*. *cuvieri* in northeastern Amazonia

Patton et al. [10] assessed the genetic divergence in *P*. *cuvieri* samples from Brazil (Amazonas and Pará), together with localities in Peru, Venezuela and French Guiana, using sequences of the cytb gene and confirmed the monophyly for eastern Amazonia, including Guianas region clade. Our phylogenetics analyses recovered a clade of *P*. *cuvieri* in northeastern of the Amazon region which appeared distinct from samples of the other Amazon regions. For *P*. *guyannensis* the haplotypes of northeastern region were recovered in a clade separated from the other samples of the distribution area of this species (Amazonas, Roraima and Venezuela). Based on the analysis of cytb and of karyotypes for *P*. *guyannensis* samples, Bonvicino et al. [28] recovered three clades in the Guiana region including one north-eastern clade (French Guiana) and two western clades (Roraima and Amazonas/Venezuela).

We inferred a late Pliocene origin for the diversification of the clade from the eastern Guianas region for *P*. *guyannensis* at approximately 2.8 Mya and early Pleistocene for *P*. *cuvieri* (2.19 Mya). In both species, however, the divergence in four (*P*. *guyannensis*) and five (*P*. *cuvieri*) lineages occurred during the early Pleistocene (Fig 2). The early Pleistocene is consistent with the intense geomorphological changes occurring in the estuary of the Amazon River, which formed its current course, and the formation of parts of the coast of Amapá [5, 72, 73]. Temperatures also decreased during the Pleistocene [74], leading to the expansion of drier vegetation and the fragmentation of forest blocks [8, 75]. Despite the relatively limited size of our study area probably these phenomena had distinct impacts on its northern and southern portions contributing to the diversification of the populations of both *P*. *cuvieri* and *P*. *guyannensis*.

In spite of the limitation of mitochondrial data, it has already been shown to be robust [76] and has long been considered as a standard for phylogeography and for assessing genetic variation within and between populations [77]. Further, high ration of copies compared to nuclear genome allows for isolation from long dead tissues and collection-preserved samples, and consequently favor work on large dataset, not requiring new field captures for fresh and/or high quality samples. Here we significantly increased the knowledge about the phylogeography of these two species Amazonian Spiny rats [29, 34] confirming the uniqueness of the western portion of the Guyana region. Our conclusions and interpretations should be confirmed by other molecular characters, complex processes of fragmentation, expansion and admixture—as can be inferred from our study—should be confirmed through the use of several and complementary types of molecular markers.

### Genetic variability and demography of *P*. *guyannensis* and *P*. *cuvieri*

Different hydrological factors, including size and course stability [10], may influence the effectiveness of a river to act as a barrier to gene flow. Previous studies suggested ther is no evidence for a major role of rivers in the genetic differentiation of most Amazonian rodents [4, 10]. For example, the spiny rats *Proechimys steerei* and *P*. *simonsi* share haplotypes on both margins of the Rio Juruá which a highly meandering river with intense seasonal flooding in western Amazonia [10, 16]. Similarly our results suggest that the Oiapoque, Araguari and Jari rivers have not acted as barriers to the dispersal of *P*. *guyannensis* and *P*. *cuvieri*, since haplotypes were shared between both margins of these rivers and included in the same genetic cluster (Figs 3 and 4). In addition, genetic distances for *P*. *guyannensis* were significantly smaller in between opposite margins than between clusters (Table 2). Moreover the permeability of riverine barriers is highly dependent on species-specific traits [78]. For example, the Oiapoque river seems to be a more efficient barrier for amphibian species that are leaf-litter dwellers or lack free-living tadpoles [79].

However our results indicate that the northeastern part of the Guianas region seems to have undergone distinct evolutionary events. We found clear genetic structuring in our sample of *P*. *guyannensis*, with an association between the haplotype network and the geographic distribution of the four clusters identified as G1, G2, G3 and G4. The diversity of *P*. *guyannensis* has been established through karyological [28, 30, 33], molecular [28, 34] and morphological studies [36, 80]. French Guiana is located entirely within the area of the Guianan Shield, this region is an ancient region and previous studies regarded it like a forest refugium of northeastern Amazonian [81]. Van Vuuren et al. [34] suggested a recent expansion based in the high number of shared haplotypes in *P*. *guyannensis* from the regions of Cayenne and Petit Saut (see Fig 1), in French Guiana. With the increase of the sampled area in northeastern Amazonia, we identified a cluster (G1) that groups samples from northern of French Guiana to Oiapoque River, with a large number of shared haplotypes. This cluster includes a haplotype (H1) that is shared with the majority of the samples from Cayenne, with one principal haplotype (H35) that forms the center of a star-like network, which may reflect recent colonization and/or are expanding population [34, 82].

In *P*. *cuvieri* we recorded also four clades, of which cluster C1 occurs in the north of our study area, in the region between the Maroni and Mana rivers in northern French Guiana. We estimated the diversification at approximately 1.76 Mya for cluster C1 in *P*. *cuvieri* and 1.68 Mya for G1 in *P*. *guyannensis* (Fig 2). These two datings, almost similar, might be the result of a process of genetic isolation in forest fragments produced by the intense changes in the configuration of the region’s vegetation that occurred during the early Pleistocene and late Pliocene [8, 83]. Cold and warm periods of the Pleistocene, isolating populations and promoting backward and forward migrations, may also have led to competitive interactions between the two species, and facilitated structuration, in a dynamic process comparable to the disturbance-vicariance hypothesis [84].

We also detected a signal of recent expansion indicated by the high degree of haplotype sharing (Fig 3) and demographic changes (Fig 5 and Table 3). The most intense demographic expansion in both *P*. *cuvieri* and *P*. *guyannensis* occurred over the past 20,000 years (Fig 5), when the climate became more favorable for mesic environments after the Last Glacial Maximum (LGM) (20,000 yr BP) [85, 86]. While there is no clear consensus on the extent to which the region’s forests were reduced or fragmented during the LGM [86], it does seem likely that the forests shrank as temperatures decreased [74, 87]. In north-eastern Amazonia, open habitats, such as dry forests and savannahs, probably expanded during the LGM [88, 89]. It seems likely that the spiny rats, *P*. *cuvieri* and *P*. *guyannensis*, benefitted from the subsequent increase in temperatures and the expansion of humid forests, as shown with their demographic expansion (Fig 5).

## Conclusions

Intense landscape changes, such as the expansion of drier vegetation and the fragmentation of forest blocks related to the decrease in temperatures during the Pleistocene, and the intense geomorphological changes occurring in the estuary of the Amazon River around this time, had distinct impacts on the northern and southern portions of eastern Guianas. This climate and landscape changes probably drivers the diversification of the two spiny rats. Consequently, *Proechimys cuvieri* and *P*. *guyannensis* populations in the eastern portion of the Guiana region were geographically structured. Apparently two events were important in the structuration of populations in both species of spiny rats, first the geomorphological changes having occurred during the early Pleistocene (having led to the different clusters in each species: Fig 2) and much later the climatic changes in Late Pleistocene (LGM) resulting in the structuration of some clusters (Fig 3 and Fig 4). Then, the clusters G1 and C1 went through a demographic expansion (Fig 5) with the end of the LGM, probably resulting in a founding effect, mainly in *P*. *guyannensis* (G1). The genetic distances recorded associated with the structured population clusters, reinforce the need for a more detailed investigation of the comparative ecology and phylogeography of both species, with special attention to *P*. *guyannensis* for testing its well-marked structuration. Likely different ecological traits, use of space, preferred resources, adaptive ability to face habitat modifications, should be described more deeply. For a more detailed systematic review of the genus, it is necessary to include specimens from throughout the distribution of *Proechimys*, employing wider genetic or genomic approaches and karyotyping.

## Supporting information

S1 Appendix Localities of the samples (tissues) shown on Fig 1, geographic coordinates (in DD°, DDD), haplotypes (H) and sample identification in parentheses. GenBank accession numbers for *Proechimys guyannensis* and *Proechimys cuvieri* are MK139156 to MK139242 and MK184542 to MK184553.
(DOCX)

S2 Appendix Phylogenetic tree obtained by Bayesian Inference with node supports. Each terminal is identified for sample name followed by country of origin, State was included in Brazilian samples. Clusters recovered by BAPS are identified after the terminals for *P*. *guyannensis* (G1, G2, G3, G4) and *P*. *cuvieri* (C1, C2, C3, C4).
(TIFF)

S3 Appendix Phylogenetic tree obtained by maximum likelihood with node supports. Each terminal is identified for sample followed by country of origin, State was included in Brazilian samples. We also indicate the state of origin in the case of Brazil. Clusters recovered by BAPS are identified after the terminals for *P*. *guyannensis* (G1, G2, G3, G4) and *P*. *cuvieri* (C1, C2, C3, C4).
(TIFF)
