# Supplementary material for: The role of Pleistocene climate change in the genetic variability, distribution and demography of Proechimys cuvieri and P. guyannensis (Rodentia: Echimyidae) in northeastern Amazonia
Source: PLoS One. 2018 Dec 17;13(12):e0206660. doi: 10.1371/journal.pone.0206660 (PMC6296739; doi:10.1371/journal.pone.0206660)
Supplement: S1 Appendix — GenBank accession numbers for Proechimys guyannensis and Proechimys cuvieri are MK139156 to MK139242 and MK184542 to MK184553. (DOCX) [file pone.0206660.s001.docx]

Supporting information

**S1 Appendix. Localities of the samples (tissues) shown on Figure 1, geographic coordinates (in DD°, DDD), haplotypes (H) and sample identification in parentheses. GenBank accession numbers for *Proechimys guyannensis* and *Proechimys cuvieri* are MK139156 to MK139242 and MK184542 to MK184553.**

| **Sites** | **Locality** | **Long** | **Lat** | **Haplotype / Sample identification** | | **GENBANK accession number** | **Reference** |
| --- | --- | --- | --- | --- | --- | --- | --- |
| **Fig. 1** |  |  |  | ***P. guyannensis*** | ***P. cuvieri*** |  |  |
| 1 | Baramita, Barima-Waini, Guiana | -60,48 | 7,36 |  | H37 (PCU2336);  H38 (PCU2340) | AY206626; AY206623 | Van Vuuren et al. 2004 |
| 2 | Sainte Anne, Saint Laurent du Maroni, French Guiana | -53,95 | 5,47 |  | H27 (M397) |  | This study |
| 3 | Saint Jean de Maroni, French Guiana | -54 | 5 |  | H33 (PCU1757) | AJ251400 | Steiner et. al. 2000 |
| 4 | Sparouine, Apatou, French Guiana | -54,03 | 5,27 | H27 (M281) |  |  | This study |
| 5 | Awala, Awala-Yalimapo, French Guiana | -53,91 | 5,73 | H20 (M302); H21 (M299); H22 (M309); H23 (M310); H24 (M312) |  |  | This study |
| 6 | Angoulème, Mana, French Guiana | -53,66 | 5,41 |  | H15 (F1101); H26 (M358) |  | This study |
| 7 | Piste de Saint Elie, Sinnamary, French Guiana | -53,03 | 5,32 |  | H24 (M267) |  | This study |
| 8 | Sinnamary, French Guiana | -53,03 | 5,12 |  | H21 (M180); H22 (M181); H23 (M183) |  | This study |
| 9 | Petit Saut, French Guiana | -53,06 | 5,06 | H33 (PCA1394, PCA1762, PCA1832, V815) | H31 (PCU1561); H32 (PCU1750) | AJ251395, AJ251396, AJ251397; AJ251399 / AY206624, AJ251403 | Steiner et. al. 2000; van Vuuren et al. 2004 |
| 10 | Trinité, French Guiana | -53,36 | 4,56 |  | H34 (PCU1833) | AJ251401 | Steiner et. al. 2000 |
| 11 | Macouria, French Guiana | -52,48 | 5,02 | H35 (PCA1990); H36 (PCA1991) |  | AY206602, AY206604 | van Vuuren et al. 2004 |
| 12 | Cayenne, French Guiana | -52,29 | 4,91 | H1 (M632, M654, M662, M673, M685, M690, M631, M699, M703, M712, M438, PCA2656, PCA2725, PCA2715, PCA3292); H2 (M635); H16 (M1551, A1038) |  | This study, AY206601, AY206608, AY206603, AY206600; this study | van Vuuren et al. 2004 |
| 13 | Piste de Bélizon, Régina, French Guiana | -52,33 | 4,42 |  | H25 (M356) |  | This study |
| 14 | Les Nouragues, Régina, French Guiana | -52, 70 | 4,09 | H46 (T2010) | H35 (PCU1856), H36 (PCU2271) | AJ251398/ AY206633, AY206625 | Steiner et. al. 2000; van Vuuren et al. 2004 |
| 15 | Pic Matecho, French Guiana | -53, 03 | 3,73 | H37 (PCA2476)/ H28 (PCA2477) | H39 (PCU2475), H40 (PCU2479) | AY206605, AY206606/ AY206627, AY206622 | van Vuuren et al. 2004 |
| 16 | Saül, French Guiana | -53, 22 | 3,62 | H3 (M821) | H41 (PCU2484), H42 (PCU2519) | ***/ AY206628, AY206629 | This study/ van Vuuren et al. 2004 |
| 17 | Mapaone river, Parque Nacional Montanhas do Tumucumaque, Laranjal do Jari, Amapá, Brazil | -54,59 | -2,19 |  | H6 (IEPA662) |  | This study |
| 18 | Saint Georges de l´Oyapock, French Guiana | -51,8 | 3,9 |  | H8 (M1550) |  | This study |
| 19 | Anotaie river, Parque Nacional Montanhas do Tumucumaque, Oiapoque, Amapá, Brazil | -52,03 | 3,22 | H9 (IEPA900) | H2 (IEPA834) |  | This study |
| 20 | Amapá Grande river, Flota do Amapá, Amapá, Brazil | -51,19 | 2,15 | H19 (IEPA3726); H41 (IEPA3725); H42 (IEPA3727) |  |  | This study |
| 21 | Anacui river, Parque Nacional Montanhas do Tumucumaque, Serra do Navio, Amapá, Brazil | -52,65 | 1,84 |  | H4 (IEPA2078); H5 (IEPA3104) |  | This study |
| 22 | Amapari river, Parque Nacional Montanhas do Tumucumaque, Serra do Navio, Amapá, Brazil | -52,49 | 1, 60 |  | H44 (IEPA503) |  | This study |
| 23 | Mutum river, Parque Nacional Montanhas do Tumucumaque, Pracuúba, Amapá, Brazil | -52,93 | 1,39 |  | H3 (IEPA874) |  | This study |
| 24 | Santo Antônio stream, Floresta Nacional do Amapá, Serra do Navio, Amapá, Brazil | -51,88 | 1,13 | H11 (IEPA668) | H7 (IEPA502) |  | This study |
| 25 | Braço stream, Flona do Amapá, Amapá, Brazil | -51,59 | 1,31 | H19 (IEPA169) | H16 (IEPA172) |  | This study |
| 26 | Falsino river, Flona do Amapá, Porto Grande, Amapá, Brazil | -51,62 | 0,97 | H25 (IEPA3506); H26 (IEPA3451) | H19 (IEPA3305, IEPA3303); H20 (IEPA3442) |  | This study |
| 27 | Tracajatuba river, Ferreira Gomes, Amapá, Brazil | -51,16 | 1,03 | H10 (IEPA2093) |  |  | This study |
| 28 | Ferreira Gomes, Araguari river, Amapá, Brazil | -51,2 | 0,88 | H17 (IEPA2364); H18 (IEPA2365); H13 (IEPA2382) |  |  | This study |
| 29 | Caldeirão Falls, Araguari river, Porto Grande, Amapá, Brazil | -51,32 | 0,86 | H12 (IEPA3367); H13 (IEPA3039); H14 (IEPA3051) |  |  | This study |
| 30 | São Bento Farm, Apurema river, Tartarugalzinho, Amapá, Brazil | -50,77 | 1,3 | H5 (IEPA2551) | H1 (IEPA2557, IEPA2562, IEPA2574) |  | This study |
| 31 | Horto Matapi, Porto Grande, Amapá, Brazil | -51,25 | 0,48 | H4 (IEPA2558); H6 (IEPA2604); H7 (IEPA2606) |  |  | This study |
| 32 | Cupixi river, Reserva de Desenvolvimento Sustentável do rio Iratapuru, Laranjal do Jari, Amapá, Brazil | -52,33 | 0,58 | H8 (IEPA899, IEPA2059) |  |  | This study |
| 33 | Vila Nova river, FE do Amapá, Mazagão, Amapá, Brazil | -52,01 | 0,46 | H43 (IEPA3705); H44 (IEPA3712); H45 (IEPA3692) |  |  | This study |
| 34 | Vila Nova river, Mazagão, Amapá, Brazil | -51,58 | 0,13 | H47 (IEPA3235) |  |  | This study |
| 35 | Santana Island, Santana, Amapá, Brazil | -51,15 | -0,07 |  | H17 (IEPA3820) |  | This study |
| 36 | Jari river, Reserva de Desenvolvimento Sustentável do rio Iratapuru, Laranjal do Jari, Amapá, Brazil | -53,11 | -0,28 |  | H43 (IEPA2081) |  | This study |
| 37 | Marinho Village, Reserva Extrativista do Rio Cajari, Laranjal do Jari, Amapá, Brazil | -52,24 | -0,56 |  | H9 (IEPA1621); H10 (IEPA1606) |  | This study |
| 38 | Itacará, Jari river, Laranjal do Jari, Amapá, Brazil | -52,68 | -0,51 |  | H18 (IEPA2740); H46 (IEPA2770) |  | This study |
| 39 | Santo Antônio Falls, Jari river, Laranjal do Jari, Amapá, Brazil | -52,52 | -0,62 | H15 (IEPA2403) | H11(IEPA2404); H13 (IEPA2072); H14 (IEPA2736); H45 (IEPA1511) |  | This study |
| 40 | Porto do Sabão, Jarí river, Almeirim, Pará, Brazil | -52,53 | -0,62 |  | H12 (IEPA2728) |  | This study |
| 41 | Jatuarana Village, Prainha, Pará, Brazil | -53,67 | -1,59 | H38 (IEPA3003); H39 (IEPA3464); H40 (IEPA2406) |  |  | This study |
| 42 | Mamiá Village, Curuá, Pará, Brazil | -55,15 | -1,55 | H29 (IEPA1804); H30 (IEPA3952); H31 (IEPA1807); H32 (IEPA3245) | H28 (IEPA1806); H29 (IEPA3192); H30 (IEPA3194) |  | This study |
| 43 | Alto Jatapu, Roraima, Brazil | -59,91 | 0,95 | CRB617 |  |  | This study |
| 44 | Lago Meduinim, rio Negro, Amazonas, Brazil | -61,28 | -1,79 |  | JLP16804 |  | This study |
| 45 | Barro Vermelho, rio Juruá, Amazonas, Brazil | -68,77 | -6,47 |  | MVZ190697 |  | This study |
| 46 | Pico da Neblina, Amazonas, Brazil | -68,63 | 0,05 | VCSV73, VCSV74 |  |  | This study |
| 47 | Amazonas, Peru | -77,82 | -3,04 |  | JLP8380 |  | This study |
| 48 | Serra do Apiaú, Roraima, Brazil | -62,42 | 2,62 | SAM03 |  |  | This study |
| 49 | Bolivar, Venezuela | -63,57 | 6,35 |  | MVZ160092 |  | This study |
