# Supplementary figures and images for: The role of Pleistocene climate change in the genetic variability, distribution and demography of Proechimys cuvieri and P. guyannensis (Rodentia: Echimyidae) in northeastern Amazonia
Source: PLoS One. 2018 Dec 17;13(12):e0206660. doi: 10.1371/journal.pone.0206660 (PMC6296739; doi:10.1371/journal.pone.0206660)

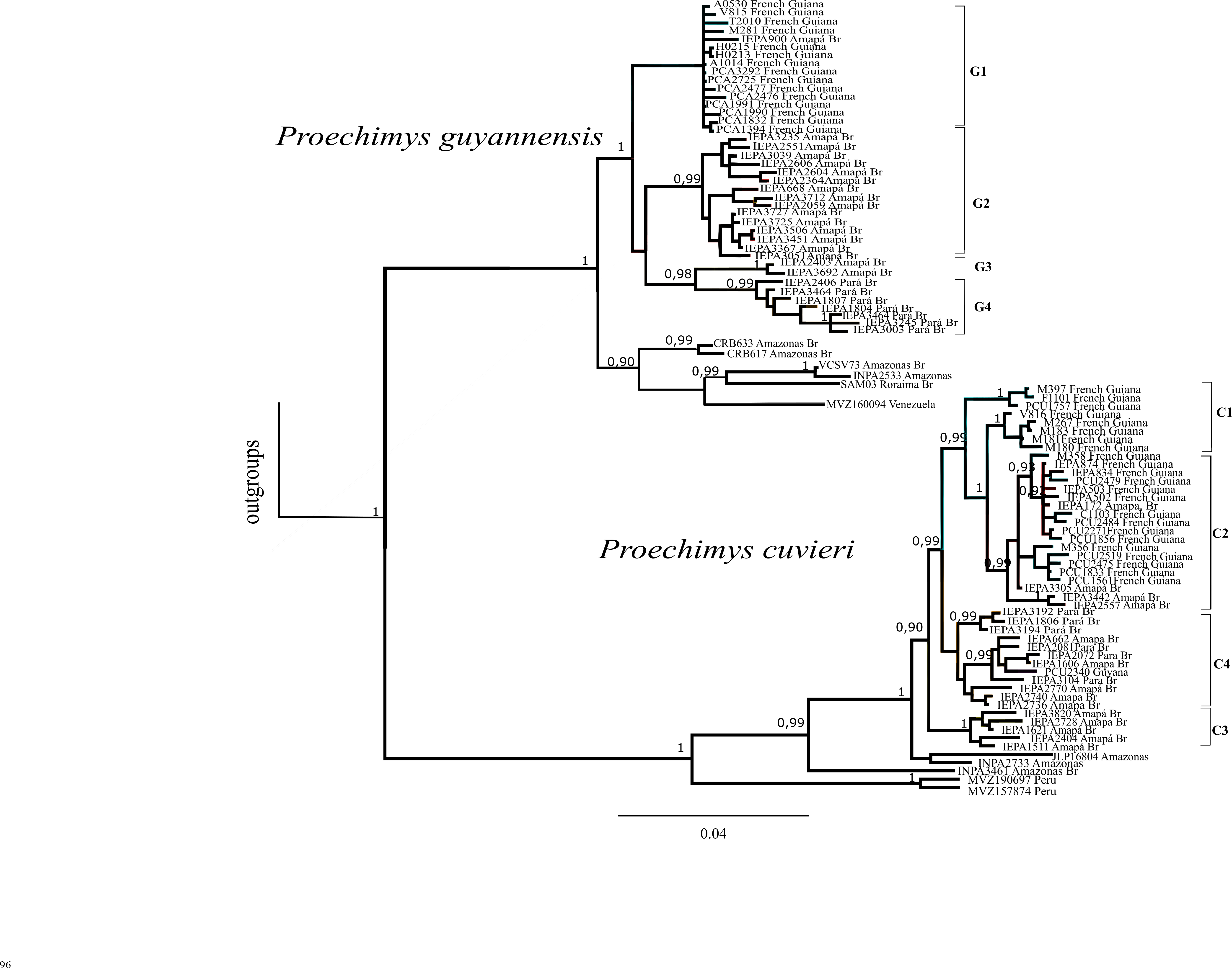

Supplement: S2 Appendix — Each terminal is identified for sample name followed by country of origin, State was included in Brazilian samples. Clusters recovered by BAPS are identified after the terminals for P. guyannensis (G1, G2, G3, G4) and P. cuvieri (C1, C2, C3, C4). (TIFF) [file pone.0206660.s002.tiff]

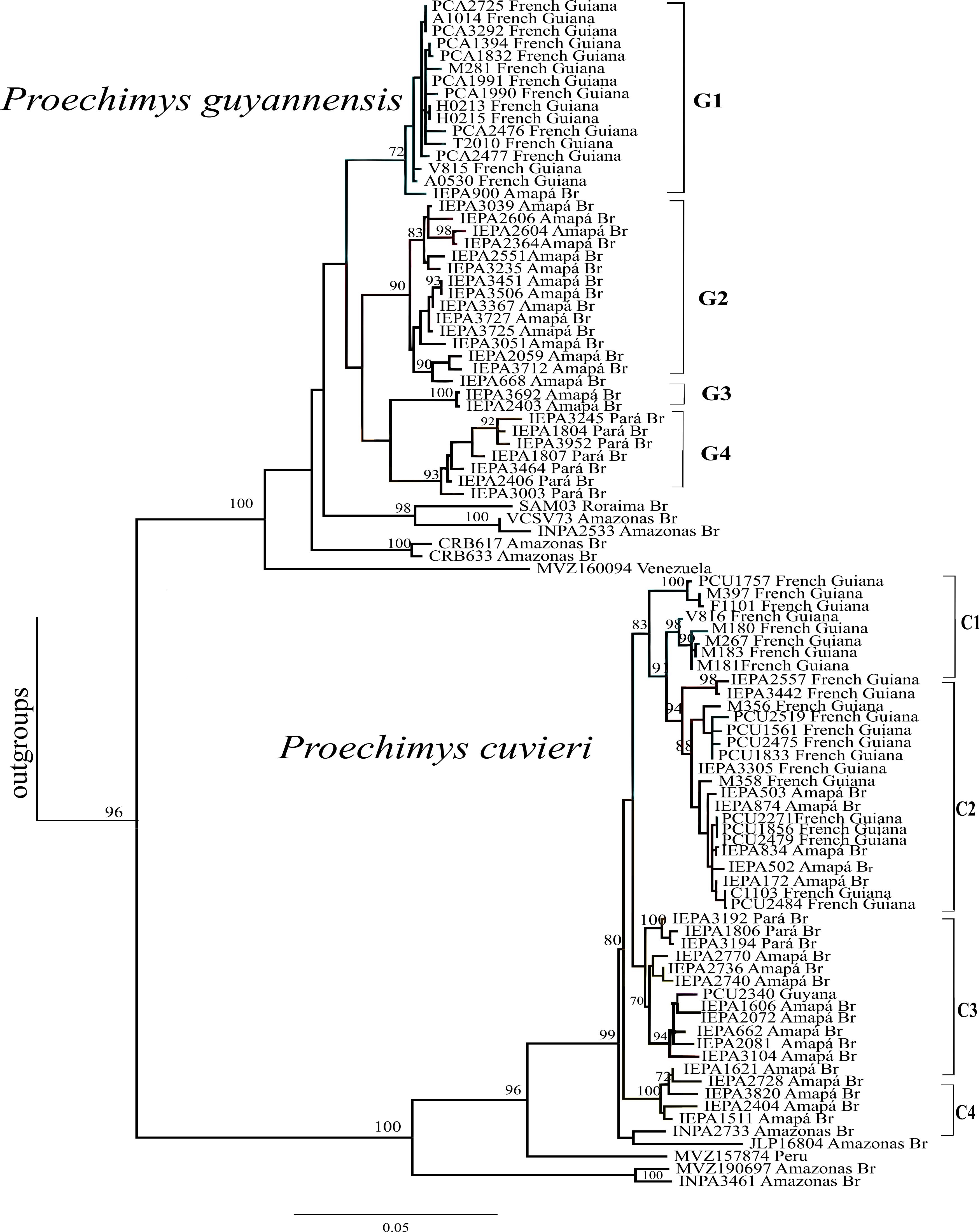

Supplement: S3 Appendix — Each terminal is identified for sample followed by country of origin, State was included in Brazilian samples. We also indicate the state of origin in the case of Brazil. Clusters recovered by BAPS are identified after the terminals for P. guyannensis (G1, G2, G3, G4) and P. cuvieri (C1, C2, C3, C4). (TIFF) [file pone.0206660.s003.tiff]
